# Can Intrinsic and Extrinsic Metacognitive Cues Shield Against Distraction in Problem Solving?

**DOI:** 10.5334/joc.9

**Published:** 2018-02-21

**Authors:** Linden J. Ball, Emma Threadgold, Anna Solowiej, John E. Marsh

**Affiliations:** 1 School of Psychology, University of Central Lancashire, Preston, UK; 2 School of Experimental Psychology, Heinrich Heine University, Düsseldorf, DE; 3 Department of Building, Energy and Environmental Engineering, University of Gävle, Gävle, SE

**Keywords:** Auditory Distraction, Intrinsic and Extrinsic Metacognitive Cues, Processing Fluency, Problem Solving, Task Engagement

## Abstract

We investigated the capacity for two different forms of metacognitive cue to shield against auditory distraction in problem solving with Compound Remote Associates Tasks (CRATs). Experiment 1 demonstrated that an intrinsic metacognitive cue in the form of processing disfluency (manipulated using an easy-to-read vs. difficult-to-read font) could increase focal task engagement so as to mitigate the detrimental impact of distraction on solution rates for CRATs. Experiment 2 showed that an extrinsic metacognitive cue that took the form of an incentive for good task performance (i.e. 80% or better CRAT solutions) could likewise eliminate the negative impact of distraction on CRAT solution rates. Overall, these findings support the view that both intrinsic and extrinsic metacognitive cues have remarkably similar effects. This suggests that metacognitive cues operate via a common underlying mechanism whereby a participant applies increased focal attention to the primary task so as to ensure more steadfast task engagement that is not so easily diverted by task-irrelevant stimuli.

Research on metacognition focuses on the processes that monitor and control cognition, both during relatively straightforward tasks, such as memorisation and knowledge retrieval, as well as during more complex tasks, such as reasoning and problem solving (see Ackerman & Thompson, 2017a, 2017b). At a finer level of conceptual analysis, *metacognitive monitoring* processes typically relate to people’s subjective assessment of how well a current task is being performed, but such monitoring can also extend both to future-oriented judgments of how well a task *will be* performed and to after-the-event judgments of how well a task *has been* performed. In the specific case of metacognitive monitoring processes about a task at hand, these judgments usually reflect fluctuating states of certainty or uncertainty about task performance and interlink with *metacognitive control* processes that are applied to initiate, terminate, or change the allocation of time, effort and cognitive resources to the task (Ackerman & Thompson, 2017a). It is widely accepted that metacognitive monitoring is based on an extensive variety of cues (for further discussion see Ackerman & Thompson, 2017a, 2017b; Thompson, 2009; Topolinski, 2017), including cues that derive from perceivable features of the task (e.g., its apparent complexity) and from one’s own experience of attempting the task (e.g., subjective ease of processing). Potentially, too, metacognitive cues might originate from sources that are peripheral to the immediate task environment, such as performance incentives or one’s inherent motivation to achieve. The research that we report in the present paper was concerned with investigating two distinct metacognitive cues: external incentives to achieve good performance outcomes and the subjective experience of task ease or difficulty.

In order to understand such metacognitive modulation of thought, researchers have drawn heavily on dual-process theories (e.g., Evans, 2007; Kahneman, 2011), which emphasise the interplay between two qualitatively distinct types of thinking. According to the recent proposals of Evans and Stanovich (2013a, 2013b), “Type 1” processes have two defining features: they are autonomous and undemanding of working-memory (a concept they use in ways that also link to notions of executive and attentional control; cf. Norman & Shallice, 1986). Type 1 processes also tend to be fast, non-conscious, intuitive and associative, but these are simply examples of what Evans and Stanovich see as being correlated features rather than defining features. “Type 2” processes, on the other hand, are defined by Evans and Stanovich in terms of relying on working-memory (including executive and attentional control) and as being focused on cognitive decoupling and mental simulation, which are critical for hypothetical thinking. Type 2 processes also tend to be slow, conscious, analytic and deliberative, but again, these are viewed as being correlated features rather than defining features.

Two key factors that have been proposed to be instrumental in catalysing a shift from Type 1 to Type 2 processing are those that we have alluded to above, that is, motivation (whether self-determined or externally-driven) and the subjective experiences of task difficulty (e.g., Alter, 2013; Alter, Oppenheimer, Epley, & Eyre, 2007; Rummer, Schweppe, & Schwede, 2016). For example, Type 2 processes are argued to become obligatory if the individual categorizes the task at hand as highly important, such as when a reward (e.g., a prospective exam result) is dependent on obtaining a high level of performance. It is also proposed that Type 2 processes can be activated if the monitoring system – as part of the metacognitive architecture – judges that a task is difficult (e.g., Bjork, Dunlosky, & Kornell, 2013; see also Thompson, 2010).

## Processing Disfluency as a Metacognitive Cue

This triggering of Type 2 thinking as a response to perceived task difficulty can itself be cued through the experience of *processing disfluency*. As a specific case in point, it has been proposed (e.g., Alter et al., 2007) that whilst easy-to-read text (e.g., presented in Arial font) engages Type 1 processing and intuitive responses, difficult-to-read text (e.g., presented in Haettenschweiler) serves as a cue that the task is difficult (and that responses based on Type 1 processes may be incorrect), thereby activating Type 2 processing. Since it is only the surface characteristics of text that are changed, processing disfluency is not necessarily tied to objectively defined task difficulty – it can simply act as an influence on subjectively perceived task difficulty (Rummer et al., 2016). Importantly, however, several influential studies (e.g., Alter et al., 2007; Diemand-Yauman, Oppenheimer, & Vaughan, 2011) have proposed that switching from Type 1 to Type 2 processes in the manner just described usually results in better task performance, producing so-called *disfluency effects*.

Disfluency effects have been observed in the context of studies on semantic illusions (Song & Schwartz, 2008), learning of class materials (Diemand-Yauman et al., 2011; French et al., 2013), cognitive biases (Hernandez & Preston, 2013), word-learning (Sungkhasettee, Friedman, & Castel, 2011), non-intuitive problem solving (Alter et al., 2007) and syllogistic reasoning (Alter et al., 2007). However, other studies have either failed to find such disfluency effects or have failed to replicate them (e.g., Carpenter, Wilford, Kornell, & Mullaney, 2013; Eitel Kühl, Scheiter, & Gerjets, 2014; Kühl & Eitel, 2016; Rummer et al., 2016; Yue, Castel, & Bjork, 2013). Arguably, the current weight of evidence would appear to undermine the generality of disfluency effects and expose their fragility (Meyer et al., 2015; Thompson et al., 2013a). To this end, boundary conditions for the manifestation of disfluency effects clearly need to be established. This paper addresses the notion that disfluency effects may be contingent on particular task conditions (cf. Kühl & Eitel, 2016; see also Alter, Oppenheimer, & Epley, 2013).

## Metacognitive Cues and Distraction Shielding

Set against a backdrop of uncertainty in relation to the robustness and replicability of disfluency effects (Thompson et al., 2013a; Meyer et al., 2015) is a small, but growing, body of work demonstrating that processing disfluency can modulate distraction by task-irrelevant speech (Halin, 2016; Halin, Marsh, Haga, Holmgren, & Sörqvist, 2014; Halin, Marsh, Hellman, Hellström, & Sörqvist, 2014; Hughes, Hurlstone, Marsh, Vachon, & Jones, 2013; Marsh, Sörqvist, & Hughes, 2015; Marsh et al., 2017). The presence of task-irrelevant speech typically disrupts performance on visually-based tasks such as proof-reading (Jones, Miles, & Page, 1990), reading comprehension (Martin, Wogalter, & Forlano, 1988) and memory for prose (Banbury & Berry, 1998). However, presenting text in a difficult-to-read font (Haettenschweiller) eliminates the disruption produced by task-irrelevant speech on the detection of semantic/contextual errors in proof-reading (Halin, Marsh, Haga et al., 2014) and memory for prose as assessed by multiple choice questions (Halin, Marsh, Hellman et al., 2014).

Similar to the perceived role of disfluency in the reasoning domain, we consider that a difficult-to-read text acts as a metacognitive cue to promote task engagement in the face of perceived difficulties. In essence, when perceived task difficulty is high, the metacognitive system instigates a compensatory upward shift in task engagement so that an individual can maintain the performance level they desire (cf. Sörqvist & Marsh, 2015; see also Eggemeier, Crabtree, & LaPointe, 1983). Furthermore, we assume that task engagement may not only be engendered by metacognitive cues such as processing disfluency inducing perceived task difficulty, but also through metacognitive cues arising from an individual’s working memory capacity, from their personal desire to succeed or from extrinsic factors affecting motivation such as incentives (Halin, Marsh, Haga et al., 2014; Hughes et al., 2013).

Akin to the supposed shifting to capacity-demanding, executive, Type 2 processes in the reasoning literature (e.g., Alter et al., 2007), it can therefore be proposed that task engagement reflects strategic cognitive control that is mediated by both intrinsic and extrinsic cues (Hughes et al., 2013). It is suggested that higher task engagement promotes a more steadfast locus of attention on the task material, thereby helping to suppress any attentional orienting toward task-irrelevant speech (Hughes et al., 2013; Marsh et al., 2015). It is further proposed that higher task engagement reduces the extent to which task-irrelevant speech interferes with deliberative cognitive activity through attenuating the processing of sound (Marsh & Campbell, 2016; Sörqvist, Stenfelt, & Rönnberg, 2012).

In contrast to disfluency effects that refer to facilitation of task performance in typical (i.e. quiet) conditions (e.g., Alter et al., 2007) due to the presence of a disfluent font, the disfluency effects reported by Halin and colleagues (Halin, 2016; Halin, Marsh, Haga et al., 2014; Halin, Marsh, Hellman et al., 2014; see also: Hughes et al., 2013; Marsh et al., 2015; Marsh et al., 2017) refer to reduced distraction by background sound when focal material was presented in a difficult-to-read font. This so-called shielding against distraction chimes with the aforementioned suggestion that disfluency effects may only emerge under specific task conditions.

## Aims and Predictions

In the experiments that we report below, which examined people’s problem-solving performance, we set out to test the prediction that resistance to distraction from task-irrelevant speech will be engendered through the presentation of task materials in either a disfluent font (i.e. an intrinsic, task-based cue; Experiment 1) or through performance incentivisation (an extrinsic, externally-based cue; Experiment 2). We also predicted that the positive effects of these metacognitive cues would be absent in the case of problem-solving performance in standard conditions wherein no speech distracter is presented.

For our reported experiments we selected the Compound Remote Associates Task (CRAT; Bowden & Jung-Beeman, 2003) as the visually-based, focal problem-solving task. A CRAT involves presenting a participant with three words (e.g., dress – dial – flower), with their task being to produce a single word (in this case “sun”) that is associated with all three words and which forms a common word or phrase when combined with each of them, either with placement before or after each word (i.e. sun-dress, sun-dial and sun-flower in the present example).

The rationale for using CRATs in our study was that previous work had confirmed their sensitivity to task-irrelevant sound manipulations (e.g., Threadgold, Marsh, & Ball, 2017), such that there is scope for metacognitive cues to facilitate in shielding against such distraction. Furthermore, the successful solution of CRATs requires an effective interplay between Type 1 and Type 2 processing. For example, it is likely that initial task responses that are based on associative (Type 1) processes such as unconscious spreading activation (Smith, Huber, & Vul, 2013) will engender strong associates of one or two words, but not all three words (e.g., dress-up and dial-up in the previous example), and will therefore be erroneous solutions. In contrast, Type 2 processes based on analytic deliberation and executive operations are likely to facilitate production of correct responses. Such Type 2 processes would include the inhibition of incorrect solution ideas that may impede production of an appropriate response via further associative processing (see Storm & Angello, 2010, for evidence for the role of inhibitory processes in CRAT solving). Type 2 processes would also involve the active manipulation of information within working memory (see Chein & Weisberg, 2013, and Ball & Stevens, 2009, for evidence that working memory proceses are involved in CRAT success).

In Experiment 1, CRATs were presented to participants either in a disfluent font (Haettenschweiler; e.g., Halin, 2016) or a fluent font (Arial). Furthermore, CRATs were either presented with or without task-irrelevant speech. If disfluency acts as a metacognitive cue to shift problem solving from Type 1 to Type 2 processes (as per Alter et al., 2007), then it is expected that more problems would be solved when the CRATs are presented in disfluent font compared to a fluent font in standard (quiet) conditions (cf. Alter et al., 2007). If, however, disfluency effects are contingent on task conditions (Kühl & Eitel, 2016) – as we predict is the case – then disfluent fonts (through engendering higher task engagement) are expected merely to shield against the disruptive effects of task-irrelevant sound on CRAT performance (see Threadgold et al., 2017), without affecting performance in standard (quiet) conditions.

## Experiment 1

### Method

#### Participants

Seventy participants took part in the experiment, either in return for course credit or for a small financial reward. Participants were staff and students at the University of Central Lancashire, UK, and were native English speakers with normal or corrected-to-normal vision and hearing. Ethical clearance was obtained prior to the experiment. Six of the 70 participants who were originally tested were excluded prior to data analysis for failing to answer any CRATs correctly in either the irrelevant speech or quiet conditions, which suggested a complete lack of task engagement. This left a total of 64 participants for the analysis (36 female and 28 male; *Range_age_* = 18 to 49 years; *M_age_* = 27 years, *SD_age_* = 8 years).

#### Design

The design was a 2 × 2 mixed design. The between-participants factor was processing disfluency with two levels (easy-to-read vs. difficult-to-read). The within-participants factor was distraction (irrelevant speech vs. quiet). The dependent variable was the number of CRATs correctly solved within two minutes out of a maximum of 10 in each condition.

#### Materials

*Compound Remote Associate Tasks*. A series of 20 CRATs were selected and divided into two sets of 10 problems each (i.e. Set A and Set B; see Appendix) so as to serve as problem-solving stimuli that could be presented in counterbalanced orders across the irrelevant speech versus quiet conditions of the within-participants distraction manipulation. Each set was matched in terms of mean solution rates (*M_Set A_* = .49, *SD_Set A_* = .26 vs. *M_Set B_* = .50; *SD_Set B_* = .26) and mean solution times (*M_Set A_* = 8.54s, *SD_Set A_* = 2.85 vs. *M_Set B_* = 8.56s; *SD_Set B_* = 2.83) using the normative data reported by Bowden and Jung-Beeman (2003). There was no significant difference across the two sets of CRATs for either the normed solution rates or the normed solution times (both *ps* > .05; refer to the Appendix for the Set A and Set B CRATs with normed solution-rate and solution-time data).

For the processing disfluency manipulation, half of the participants received the CRAT problems presented in Arial 12-point font (easy-to-read) and half of the participants received the problems in 12-point Haettenschweiler font (difficult-to-read). Haettenschweiler was selected because prior studies (e.g., Diemand-Yauman et al., 2010; Halin, 2016; Halin, Marsh, Haga et al., 2014, Experiment 1; Lehmann, Goussios, & Seufert, 2016) have adopted this font to promote processing disfluency. Moreover, task difficulty is typically rated as higher when materials are presented in Haettenschweiler font (e.g., Halin, Marsh, Hellman et al., 2014, Experiment 1).

*Irrelevant speech*. A transcript of a story was used as irrelevant speech for the task-irrelevant speech condition. The story was based on a mythical civilization known as “Ansarien” and was read in English by a male, native English speaker. The Swedish language version of this irrelevant speech has been used previously in studies of distraction by irrelevant speech (e.g., Halin, Marsh, & Sörqvist, 2015). The irrelevant speech transcript lasted 7 minutes in total, of which 2 minutes was used for the experiment. The irrelevant speech was delivered to a participant via a set of headphones from a PC computer at approximately 60–65 dB(A).

#### Procedure

Participants read an information sheet and signed a consent form prior to participating in the experiment. The experiment took place during a single 10 minute session in a quiet laboratory in the School of Psychology at The University of Central Lancashire. The presentation order of CRAT Set A and CRAT Set B was fully counterbalanced across participants, as was the conjunction of CRAT sets with either irrelevant speech or quiet. To ensure task compliance, the instructions indicated that participants would receive a “special bonus prize” for scoring an average of 20% correct across all CRATs. This instruction was presented at the beginning of each set of CRATs.

For each set of CRATs, the participants were given a maximum of two minutes to answer the problems in any order they wished. For the irrelevant speech condition, participants were presented with irrelevant speech played on the computer via headphones and were instructed to ignore the speech and to complete the problems. At the end of testing, each participant was awarded a 4GB USB pen drive as their special bonus prize, regardless of the average score achieved on the CRATs. Each participant was fully debriefed at the end of the experiment and thanked for their participation time.

## Results and Discussion

**Figure 1:**
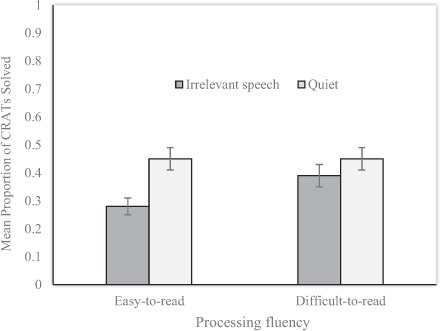
Mean proportion of CRATs solved correctly for the irrelevant speech and quiet conditions as a function of processing disfluency (error bars depict the standard error of the mean).

The dependent variable was the proportion of CRATs solved correctly out of 10 problems in each condition. The mean proportion of CRATs solved for the irrelevant speech and quiet conditions as a function of processing disfluency is shown in Figure 1. A 2 × 2 mixed analysis of variance (ANOVA) was conducted on the mean proportion data. The within-participants factor was distraction (irrelevant speech vs. quiet), with processing disfluency (easy-to-read vs. difficult-to-read) as the between-participants factor. The ANOVA revealed a main effect of distraction, *F*(1, 62) = 17.21, *MSE* = .025, *p* < .001, *η_p_^2^* = .022, but not of processing disfluency, *F*(1, 62) = 1.45, *MSE* = .06, *p* = .230, *η_p_^2^* = .023. The distraction × processing disfluency interaction also failed to meet conventional levels of significance, *F*(1, 62) = 2.93, *MSE* = .025, *p* = .091, *η_p_^2^* = .045.

A full-blown unpacking of all simple main effects in not warranted because of the absence of any protection from a significant interaction, with the lack of this protection potentially increasing the familywise Type 1 error rate. Nevertheless, we emphasize here that our *a priori* predictions were fundamentally set up to allow for the pursuit of a more limited set of planned comparisons. Such planned comparisons are entirely warranted despite the lack of a reliable interaction (e.g., see Roberts & Russo, 1999, pp. 181–193), especially given the intention to focus on just two comparisons rather than the full set of four simple main effects analyses that are traditionally needed to examine a significant 2 × 2 interaction effect.

The two planned comparisons that we were interested in with respect to our predictions concerning CRAT performance are as follows. First, we wished to test for a significant improvement in the difficult-to-read-font condition versus the easy-to-read-font condition when irrelevant speech is present, since this would support our prediction that processing disfluency can act as a metacognitive cue to increase task engagement and thereby shield against the negative effects of distraction. Second, it is also necessary to test for a significant improvement in the difficult-to-read versus easy-to-read-font conditions when participants are working in quiet conditions, since a reliable effect here would indicate that processing disfluency is not merely able to act specifically as a metacognitive cue that can shield against distraction, but can act more generally to benefit problem solving via the triggering of enhanced Type 2 processing (cf. Alter et al., 2007). We note that our own expectation based on our previous review of the literature was that this latter effect would be unreliable, with processing disfluency only showing a benefit in terms of shielding against the disruptive effects of task-irrelevant speech.

Before reporting the outcome of these two planned comparisons we note the importance of using a pooled mean squared error estimate from the omnibus ANOVA in determining the *F* ratio to test each treatment effect of processing disfluency (i.e. in relation to the irrelevant-speech conditions and the quiet conditions). Deriving this pooled mean squared error estimate, however, is challenging because of the absence of a single pooled error term in the mixed ANOVA output derived from SPSS, which we used for our data analyses. We therefore needed to take the error terms from our mixed ANOVA output and calculate the pooled error term using a manual procedure proposed by Howell (2002, pp. 490–493). Conducting the requisite procedure was greatly simplified by using an Excel spreadsheet calculator that has helpfully been made openly accessible by Baguley (2006, July 10).

Our first planned comparison revealed a significant simple main effect of processing disfluency when irrelevant speech was present, *F*(1, 107.33) = 3.92, *p* = .05. This significant effect attests to the way in which processing disfluency can mitigate the detrimental impact of distraction caused by irrelevant speech on solution rates for CRATs. This finding is consistent with the notion that perceived task difficulty – engendered by processing disfluency – increases focal task engagement so as to shield against distraction (e.g., Halin, Marsh, Hellman et al., 2014). Our second planned comparison revealed that the simple main effect of processing disfluency in quiet conditions was not significant, *F*(1, 107.33) = 0.002, *p* = .961. This result indicates that an intrinsic metacognitive cue in the form of processing disfluency has no significant effect on problem solving success in standard conditions (quiet), thereby conceptually replicating prior studies (Meyer et al., 2015; Thompson et al., 2013a, 2013b) that have also failed to find disfluency effects with problem solving tasks.

One possible explanation for the predicted distraction-shielding effect that we have observed from the use of a difficult-to-read font for problem presentation is that the sensory-encoding load enforced by processing disfluency potentiates a “blocking” mechanism that prevents attentional shifts toward the task-irrelevant speech. This blocking mechanism may also curtail the processing of the background sound, thereby reducing its semantic analysis and thus the disruption this may confer to deliberative semantic processing within the focal, primary task (Sörqvist & Marsh, 2015; Marsh & Campbell, 2016).

Essentially, this aforementioned blocking account is a task-intrinsic, motivation-based explanation: the blocking mechanism is enhanced by the disfluency within the focal task, thus motivating a strategic narrowing of the attentional focus. However, it is unknown whether extrinsic motivation, that is, motivation induced by incentives external to the parameters of the task itself, also promotes the blocking of irrelevant speech. Although extrinsic motivation has been shown to offset the impact of psychological fatigue in sustained attention tasks (Davies & Parasuraman, 1982), surprisingly little research has examined whether extrinsic motivation also interacts with susceptibility to attentional distraction. One exception is a study by Engelmann, Damaraju, Padmala, and Pessoa (2009), which found that incentives can prevent task-irrelevant sound from disrupting ongoing task performance. Our view of this effect is that an incentive can act as a metacognitive cue that enhances task engagement through increasing motivation. Therefore, we expect incentives to produce a similar reduction in distraction during problem solving with CRATs as the disfluency manipulation in Experiment 1. We also note that within the reasoning literature, higher motivation is generally thought to also favour Type 2 deliberative processing (e.g., Alter et al., 2007; Rummer et al., 2016). Therefore, just as disfluent fonts would be expected to improve CRAT performance under standard (quiet) conditions, so too should motivation. In other words, both disfluency and motivation should act as metacognitive cues to engender a shift from Type 1 to Type 2 processes that are more capable of yielding CRAT solutions.

In Experiment 2, we sought further support for the notion that disfluency effects are highly condition-specific. Moreover, we tested our hypothesis that higher motivation will act as an extrinsic metacognitive cue that will increase task engagement and shield CRAT problem solving against distraction (as in Experiment 1). This experiment also permitted a further test of the notion that higher motivation results in increased attention-demanding, Type 2 processing over shallower, Type 1 processing (e.g., Alter et al., 2007; Rummer et al., 2016). If this is the case, then we would expect more problems to be solved in the standard (quiet) conditions when a high incentive for good performance was offered relative to a low incentive.

To test these predictions, participants attempted to solve CRATs in the presence of task-irrelevant speech or in quiet conditions. Furthermore, participants in the high-incentive condition were told that they would receive a reward if they obtained 80% correct responses on the CRAT problems (cf. Eysenck, 1983; Leotti & Wager, 2010) while participants in the low incentive condition were told they would receive a reward if they scored 20% correct responses on the same problems.

## Experiment 2

### Method

#### Participants

Seventy-one participants took part in the experiment, either in return for course credit or for a small financial reward. Participants were staff and students at the University of Central Lancashire, UK, and were native English speakers with normal or corrected-to-normal vision and hearing. None had taken part in Experiment 1. Ethical clearance was obtained prior to the experiment. Three of the 71 participants who were originally tested were excluded prior to data analysis for failing to answer any CRATs correctly in either the irrelevant speech or quiet conditions. This left 68 participants for the analysis (41 female and 27 male; *Range_age_* = 18 to 45 years, *M_age_* = 25 years, *SD_age_* = 8 years).

#### Design and Procedure

The design and procedure were the same as Experiment 1 with the exception that the between-participants factor was incentive (20% vs. 80% correct in the task). The incentive instruction was provided at the beginning of each problem set to reinforce its relevance. Participants in the low-incentive condition (the same as Experiment 1) were told that if they achieved greater than 20% of the CRATs correct overall then they would receive a special bonus prize, whilst participants in the high-incentive condition were told that they needed to achieve 80% to receive the bonus prize. As with Experiment 1, this prize was a 4 GB USB pen drive. All participants received the bonus prize regardless of their performance on the task and were fully debriefed at the end of the experiment.

## Results and Discussion

**Figure 2:**
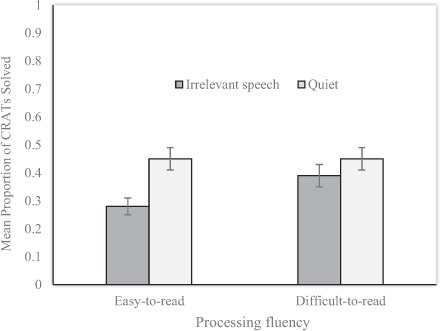
Mean proportion of CRATs solved correctly for the irrelevant speech and quiet conditions as a function of incentive (error bars depict the standard error of the mean).

The dependent variable was the proportion of CRATs solved correctly out of 10 problems in each condition. The mean proportion of CRATs solved for the irrelevant speech and quiet conditions as a function of incentive can be seen in Figure 2. A 2 × 2 mixed ANOVA was conducted on the mean proportion data, with the within-participants factor being distraction (irrelevant speech vs. quiet) and the between-participants factor being incentive (average 20% correct vs. average 80% correct). The ANOVA revealed a main effect of distraction, *F*(1, 66) = 6.49, *MSE* = .028, *p* = .013, *η_p_^2^* = .089, but no main effect of incentive, *F*(1, 66) = 2.38, *MSE* = .06, *p* = .13, *η_p_^2^* = .035. The distraction × incentive interaction was also non-significant at conventional levels, *F*(1, 66) = 3.36, *MSE* = .028, *p* = .071, *η_p_^2^* = .048.

As with Experiment 1, we were only concerned with conducting specific planned comparisons for the data arising from Experiment 2, which was a legitimate analysis approach despite the lack of any protection offered because of an unreliable interaction effect. The two planned comparisons that we were interested in with respect to our predictions concerning CRAT performance are as follows. First, we wanted to test for a significant improvement in the high incentive condition versus the low incentive condition when irrelevant speech was present, since this would support our prediction that incentive can act as an extrinsic metacognitive cue that can protect problem solvers from distraction. The relevant planned comparison revealed a significant simple main effect of processing incentive when irrelevant speech was present, *F*(1, 117.13) = 5.45, *p* = .021, with the high incentive condition mitigating the effects of distraction seen in the low incentive condition. For our second planned comparison, we wished to test for a significant improvement for high-incentive versus low-incentive instructions in the quiet conditions, since if this effect was reliable it would indicate that incentivization can act to benefit problem solving by triggering enhanced Type 2 processing. The simple main effect of incentive in the quiet conditions was, however, non-significant, *F*(1, 117.13) = 0.045, *p* = .832.

In sum, Experiment 2 unequivocally shows that incentive, an extrinsic metacognitive cue, like the intrinsic metacognitive cue of processing disfluency (Experiment 1), failed to benefit problem solving in overall terms. This result adds to the growing literature base demonstrating that metacognitive cues do not benefit problem solving in a straightforward fashion, if at all (Meyer et al., 2015; Thompson et al., 2013a, 2013b). In contrast, a high incentive clearly reduced the typical depression of CRAT solution rates produced by the presence of a speech distracter. This is consistent with the results of Experiment 1 and the general notion that motivation – engendered by offering a high incentive for good task performance – promotes higher task engagement that consequently reduces distraction (e.g., Halin, Marsh, Hellman et al., 2014).

## General Discussion

The findings of Experiments 1 and 2 support our suggestion that disfluency and incentive can act, respectively, as intrinsic and extrinsic metacognitive cues that serve to upregulate task engagement (see also Halin, 2016; Hughes et al., 2013; Marsh et al., 2015). The increase in task engagement may enhance “blocking”, that is, the capacity to avoid shifting attention to attention-diverting material (Sörqvist & Marsh, 2015). An alternative (or additional) possibility is that high task engagement produces sensory gating, whereby the processing of the sensory input is reduced (Marsh & Campbell, 2016; Sörqvist & Marsh, 2015). Accordingly, the contents or changes within the sound that may divert attention fail to do so because they are undetected (or are no longer as salient).

The current findings also support the view that the appearance of disfluency effects may hinge on the presence of specific task conditions. In the current study, disfluency effects manifest as a shield against distraction. The results of Experiments 1 and 2 are incompatible with the view that disfluency and higher incentives trigger attentionally-demanding, analytic, Type 2 processes, thereby yielding superior performance (e.g., Alter et al., 2007; Rummer et al., 2016). Out of kilter with this expectation, disfluency (Experiment 1) and a high incentive (Experiment 2) did *not* give rise to better performance in the standard (quiet) conditions. While this finding coheres with recent failures to replicate disfluency effects in the context of problem solving (Meyer et al., 2015; Thompson et al., 2013a) and cognition generally (for a review, see Kühr & Eitel, 2016), it is possible that moderator variables are at play. In the current study, the metacognitive cues were manipulated between-participants. It is possible that metacognitive triggering of Type 2 processes depends on the experience of *relative disfluency* between items or conditions (see Wänke, & Hansen, 2015) that only operates in within-participant designs. However, previous studies that directly manipulate whether the disfluency manipulation is within or between participants have failed to find (or replicate) beneficial disfluency effects in either design (Yue et al., 2013; Rummer et al., 2016; see also Thompson, Therriault & Newman, 2016).

Another possible reason for the failure to find disfluency effects in the standard conditions of Experiment 1 is that whilst the disfluency manipulation was successful in prompting Type 2 processing and analytical thought, it did not improve problem-solving accuracy (Alter et al., 2013). Previous research (e.g., Thompson et al., 2013a) has shown that disfluency can increase study time in the absence of increasing task accuracy. In our experiments, an overall time-limit of 2 minutes was imposed wherein participants were required to solve as many of the 10 simultaneously-presented CRAT items as possible. It is possible that disfluency promoted different time-sharing strategies such that individuals spent more time on each problem before progressing to another. Moreover, participants may have adopted different solution strategies achieving the same number of correct responses but through different processes (see Chein & Weisberg, 2013). Future studies could clarify these latter issues by soliciting strategy reports from participants regarding whether they solved each CRAT via insight (a Type 1 process) or analysis (a Type 2 process), so that any strategic shifts in processing approach engendered by disfluency might be detected.

We also note that at first blush, the benefit of disfluency in shielding against distraction (Experiment 1) would appear to be consistent with “load theory” (e.g., Lavie & DeFockert, 2003). Here the shielding against distraction could be attributable to a passive by-product of the depletion of an attentional resource dedicated to perceptual processing. In other words, disfluency acts as a perceptual load that leaves fewer attentional resources for processing task-irrelevant distracters, therefore mitigating distraction. However, this account seems unlikely given that disfluency is more akin to a sensory load than a perceptual load. According to load theory, distraction is increased rather than reduced with high sensory load (Lavie & De Fockert, 2003). It would seem that this heightened distraction coupled with the cognitive load required to solve CRAT problems through attentionally-demanding Type 2 processes (Alter et al., 2007), should give rise to poorer, not better performance in the disfluent-font plus irrelevant-speech condition compared to the fluent-font plus irrelevant-speech condition, which is the opposite of what was found in Experiment 1.

Another consideration is that task-irrelevant speech induces cognitive load. Under such load, Type 1 reasoning is less likely to be corrected (e.g., Bless & Schwartz, 1999). If this is the case then the persistence of Type 1 processing could yield fixation on candidate responses that are associates of one item, but not all three, and thus incorrect solutions. It is thus possible that disfluency and high incentives offset the cognitive load imposed by task-irrelevant speech, although it is far from clear what mechanism might underpin this offsetting of cognitive load. Furthermore, it is noteworthy that previous evidence has been obtained (see Jones, Marsh, & Hughes, 2012) that runs counter to the idea that the presence of task-irrelevant speech can act as a cognitive load.

A further possibility is that metacognitive cues cause shifts from Type 1 to Type 2 processing in both standard (quiet) and task-irrelevant speech conditions, and that this modulates vulnerability to a particular *variety* of distraction (e.g., attentional diversion vs. interference-by-process). In other words, Type 1 processes may be more vulnerable to one form of distraction than another. However, this proposal is highly speculative. Certainly one idea that can be easily dismissed is that the presence of task-irrelevant speech induces processing disfluency through increasing processing difficulty (whether actual or perceived), thereby activating Type 2 processing (cf. Mehta et al., 2012). If this was the case one would expect that task-irrelevant speech should serve to enhance CRAT solutions (as background white noise did in the study by Mehta et al., 2012) rather than impairing CRAT solutions, as seen in our present experiments. In addition, a high incentive (Experiment 2) had the same effect as disfluency (Experiment 1), which appears to align better with the idea that both factors act as metacognitive cues that trigger greater top-down cognitive control over attention (e.g., Hughes et al., 2013). In conclusion, we propose that intrinsic and extrinsic metacognitive cues trigger task engagement that can usefully shield against the disruptive effect of task-irrelevant background speech.

## Data Accessibility Statement

Anonymized data for the two experiments reported in this article may be found at: http://doi.org/10.17030/uclan.data.00000149.

## Additional File

The Additional file for this article can be found as follows:

10.5334/joc.9.s1 Appendix A. CRAT – answer – normed mean solution rate (%) and normed mean solution time (s).

## Author Information

The research reported in this article was supported by funding from the British Academy and Leverhulme Trust that was awarded to John E. Marsh, Linden J. Ball and Emma Threadgold (Grant No: SG162930). The data for Experiments 1 and 2 are openly available at: https://doi.org/10.17030/uclan.data.00000149.
