# Supplementary material for: Can Intrinsic and Extrinsic Metacognitive Cues Shield Against Distraction in Problem Solving?
Source: J Cogn. 2018 Feb 21;1(1):15. doi: 10.5334/joc.9 (PMC6634472; doi:10.5334/joc.9)
Supplement: Appendix A. — CRAT – answer – normed mean solution rate (%) and normed mean solution time (s). [file joc-1-1-9-s1.pdf]

Appendix A

CRAT - *answer - normed mean solution rate (%) and normed mean solution time (s)*

Compound Remote Associates - SET A

cry / walker / face	*baby*	*(56%, 8.56s)*
health / taker / child	*care*	*(70%, 7.38s)*
sense / wealth / place	*common*	*(21%, 14.53s)*
cane / daddy / free	*sugar*	*(73%, 8.79s)*
spoon / cloth / time	*table*	*(18%, 10.18s)*
hard / bottom / garden	*rock*	*(14%, 8.21s)*
flag / north / position	*pole*	*(92%, 4.77s)*
flake / mobile / fall	*snow*	*(57%, 10.75s)*
artist / fire / route	*escape*	*(42%, 7.53s)*
stick / maker / point	*match*	*(46%, 4.78s)*

Compound Remote Associates - SET B

egg / collar / wash	*white*	*(47%, 4.19s)*
head / shade / post	*lamp*	*(17%, 8.18s)*
cover / arm / wear	*under*	*(18%, 10.18s)*
dust / cereal / toilet	*bowl*	*(71%, 7.22s)*
worm / shelf / mark	*book*	*(94%, 5.27s)*
pea / shell / chest	*nut*	*(24%, 14.05s)*
pain / serial / whale	*killer*	*(75%, 10.42s)*
date / alley / fold	*blind*	*(56%, 8.61s)*
gun / puff / room	*powder*	*(58%, 10.13s)*
age / mile / sand	*stone*	*(43%, 7.36s)*
